# Psychopathic Disorder Subtypes Based on Temperament and Character Differences

**DOI:** 10.3390/ijerph16234761

**Published:** 2019-11-27

**Authors:** J. Nicolás I. Martínez-López, María-Elena Medina-Mora, Rebeca Robles-García, Eduardo Madrigal, Francisco Juárez, Carlos-Alfonso Tovilla-Zarate, Cosette Reyes, Nadja Monroy, Ana Fresán

**Affiliations:** 1 Clinical Epidemiology Laboratory, Clinical Research Directorate, Ramón de la Fuente Muñiz National Institute of Psychiatry, Mexico City 14370, Mexicocosette@imp.edu.mx (C.R.); 2 Center of Research on Global Mental Health, Department of Innovation and Global Health, Directorate of Epidemiological and Psychosocial Research, Ramón de la Fuente Muñiz National Institute of Psychiatry, Mexico City 14370, Mexico; medinam@imp.edu.mx (M.-E.M.-M.); reberobles@imp.edu.mx (R.R.-G.); 3 General Directorate of the Ramón de la Fuente Muñiz National Institute of Psychiatry, Mexico City 14370, Mexico; eduardo.madrigal@imp.edu.mx; 4 Directorate of Epidemiological and Psychosocial Research, Ramón de la Fuente Muñiz National Institute of Psychiatry, Mexico City 14370, Mexico; 5 Multidisciplinary Academic Division, Universidad Juárez Autónoma de Tabasco, Comalcalco, Tabasco 86040, Mexico; alfonso_tovillaz@yahoo.com.mx; 6 Master in Public Mental Health, National Autonomous University of Mexico, Mexico City 04510, Mexico; nadmovi@gmail.com

**Keywords:** psychopathy, antisocial, narcissistic, temperament, character

## Abstract

The concept of psychopathy has shifted from people who commit crimes to those with a particular personality and deviant behaviors. Although antisocial personality disorder is associated with psychopathy, it also seems common in individuals with narcissistic personality traits. Psychopathy may be the expression of earlier, persistent patterns of individual characteristics as personality. The psychobiological model of personality can be useful for determining whether the expression of psychopathy differs in accordance with personality dimensions and specific personality disorders. The aim was to compare temperament and character dimensions between individuals with psychopathy with comorbid predominant antisocial or narcissistic personality traits and control subjects and to determine which dimensions distinguish these groups. Control subjects (*n* = 80) and individuals with psychopathy (*n* = 80) were assessed using the Psychopathy Checklist-Reviewed, the Structured Clinical Interview for DSM-IV Axis II disorders and the Temperament and Character Inventory-Revised. Reward dependence and Self-Directedness distinguish psychopathic individuals with predominant narcissistic personality traits whereas Novelty Seeking and Self-Transcendence characterize those with antisocial personality traits. Individuals with antisocial or narcissistic psychopathy could be identified by their temperament and character traits. The expression of psychopathy differed in accordance with biologically based, environmentally shaped personality traits.

## 1. Introduction

Psychopathy is a complex mental health construct. Definitions [1] have shifted from people who commit multiple crimes, to those with a particular combination of personality traits and socially deviant behaviors [2]. This important transition in the definition of psychopathy reflects a growing body of research in the area with the use of clear conceptual parameters operationalized in an objective assessment instrument, the Psychopathy Checklist revised [3,4,5], currently the gold standard for the assessment of psychopathy.

The construct of psychopathy has been used in the criminal justice system as well as in clinical settings and other scenarios [6,7]. However, it has been conceptualized based primarily on the presence of antisocial behaviors [8,9,10,11], which might not capture the phenomenon in a deeply and complete manner (see for example: Cleckley’s original categorization for psychopathy, which includes additional criteria not exclusively related to antisocial deviant behaviors [12]). Although antisocial personality disorder is the most frequent psychiatric entity associated with psychopathy, there is evidence that some individuals with psychopathy do not particularly express antisocial activities or aggressive traits typically associated to this personality disorder. Instead, these individuals may exhibit indifference to others’ feelings, can be effective at conning and manipulating others and come off as likable in social interactions. Their deviant behavior is only recognized over time [13]. Furthermore, some of the latter traits may resemble characteristics of other personality disorders, particularly narcissism [14]. Thus, it is generally accepted that psychopathy is not a single construct defined by a unique personality disorder such as antisocial personality disorder and should therefore be assessed in accordance to the presence of other personality disorders’ traits and with the use of structural models of personality. The study of any personality construct relies on identifying its underlying dimensionality and psychopathy is not the exception. Psychopathy may be the expression of earlier, persistent patterns of individual characteristics [15], such as personality traits and mental health factors, since they can shape the way psychopathy is externalized. An example of this approach is the proposal by Christopher J. Patrick [16,17] who conceptualizes psychopathy as a syndrome characterized by three prominent components: disinhibition, boldness and meanness, all interrelated at some level considering personality, psychopathology and neurobiological correlates, but able to define psychopathy in its varying manifestations.

There are various theoretical approaches to assess personality traits, such as the psychobiological model of personality proposed by Robert C. Cloninger [18,19,20]. This model differentiates between temperament and character dimensions of personality. Temperament considers the respective contribution of biologically based, partially inherited dimensions that are relatively stable throughout life: novelty seeking (NS) is characterized by the tendency to respond impulsively to novel stimuli through active avoidance of frustration and is considered to be mediated by a dopaminergic behavioral activation system; harm avoidance (HA) reflects the tendency to inhibit responses to aversive stimuli, leading to the avoidance of punishment or non-reward and is hypothesized to be regulated by serotoninergic behavioral inhibition system; reward dependence (RD) is defined as the tendency towards positive attachment and response to reward signals and is regulated by a noradrenergic behavioral maintenance subsystem; and persistence (PS) reflects the tendency to persevere despite frustration and fatigue based on resistance to extinction of reinforced behavior. Character refers to environmentally shaped processes related to self-concepts, values and goals and is measured by three dimensions: self-directedness (SD), which refers to the ability of an individual to control, adapt, and regulate their behavior to adapt to the demands of a situation in accordance with their goals; cooperativeness (CO), which reflects individual differences in the acceptance of other people; and self-transcendence (ST), regarded as identification with everything, conceived as parts of an essential, unified whole. Among the investigations carried out in individuals with psychopathy using the psychobiological model of personality, the Dark Triad Theory of psychopathy [21] should be highlighted. This theory proposes that people’s malevolent character is represented by three main dimensions named Machiavellianism, narcissism and psychopathy, dimensions that have shown important associations with the character dimensions of the psychobiological model of personality [22]. Despite its usefulness, it is possible that the Dark Triad does not fully consider the expression of psychopathy as no information regarding its association with temperament traits is available but should be considered. Therefore, we believe that the assessment of both temperament and character dimensions can provide more extensive information of the expression of psychopathy, reflecting a possible range of psychopathic disorders [23] characterized by differences in personality traits, in particular predominantly antisocial and predominantly narcissistic traits.

The aim of the present study was to compare temperament and character dimensions between individuals with psychopathy with comorbid predominant antisocial or narcissistic personality traits and control subjects and to determine which dimensions distinguish these groups. We decided to include these three groups as some personality traits may be similar among individuals with psychopathy and control subjects. This may in turn, reflect the various behavioral expressions of psychopathy based on individual differences related to personality traits. We hypothesized that: (a) individuals with psychopathy with predominantly narcissistic personality traits would show more similarities in their temperament and character dimensions with control subjects, while more pronounced differences would be observed in individuals with psychopathy with predominantly antisocial personality traits, and (b) novelty seeking, self-directedness, and cooperativeness scores would be able to discriminate between individuals with psychopathy with predominantly antisocial or narcissistic personality traits.

## 2. Materials and Methods

This study was approved by the Ethics and Scientific Committees of the Ramón de la Fuente Muñiz National Institute of Psychiatry (INPRFM) in Mexico City. All individuals took part voluntarily after they had received a comprehensive explanation of the nature of and procedures for the study and signed a written informed consent form to participate.

### 2.1. Participants

Individuals with psychopathy: Individuals from the general population were invited through flyers posted at the INPRFM, through the INPRFM website and social networks, which mainly included information related to narcissistic and antisocial personality traits (for example: Do people consider you emotionally cold? Are you extremely confident of yourself? Do you acknowledge not regretting anything?). Information about the protocol was given to the clinical psychiatrists of the Borderline Personality Disorder Clinic (BPD). Individuals were invited to participate in the study if during the initial assessment performed by the Clinic, clinicians identify antisocial or narcissistic personality traits or psychopathic traits. All those recruited were assessed using the Psychopathy Checklist-R (PCL-R) [24,25] and the Structured Clinical Interview for DSM-IV Personality Disorders (SCID-II) [26] for predominantly antisocial (PsyAP) or narcissistic (PsyNP) personality traits. A total of 190 individuals were evaluated. From these, 51.0% (*n* = 97) did not met criteria for psychopathy, with a PCL-R total score ≤23 and 13 subjects (6.8%) withdrew their consent to participate during the assessment with the SCID-II. A final sample of eighty individuals was recruited from the general population and the BPD Clinic of the INPRFM. Axis-I disorders, according to DSM-5 diagnostic criteria [8], were registered in accordance to the clinical interview and procedures performed at the BPD Clinic and for subjects recruited from the general population, from an interview performed by a clinical psychiatrist using DSM-5 diagnostic criteria. None of the respondents with psychopathy were on any prescribed medication (e.g., antidepressants or benzodiazepines) at the time of the assessment nor did they meet any additional personality disorder criteria.

Controls: A total of 80 controls paired by age and gender were included in this study. All respondents were interviewed by a clinical psychiatrist and screened for DSM-5 Axis-I disorders, with the PCL-R for psychopathy and the SCID-II for Axis-II disorders. Potential respondents with an Axis-I disorder, an Axis-II disorder or a score ≥23 on the PCL-R were excluded.

### 2.2. Measurement Instruments

Psychopathy Checklist-R (PCL-R): this is a clinical rating scale designed to assess personality traits that define psychopathy [24]. It comprises 20 items scored on a 3-point Likert scale (0 = not present to 2 = item definitely present), with a total possible score of 40, indicative of prototypical persons with psychopathy. The PCL-R has two main factors: Factor 1—interpersonal and affective, designed to assess selfish, callous personality and Factor 2—lifestyle and antisocial, which measures socially deviant behavior and past criminality. A cutoff score of 23 was used to identify people with prototypical psychopathic characteristics [24,27]. Construct validity of the instrument, obtained by an exploratory factor analysis, reports a two-factor solution, the same as the originally proposed, with an adequate internal consistency for both factors (Factor 1 Cronbach’s alpha = 0.87 and Factor 2 Cronbach’s alpha = 0.85) [25]. The PCL-R was administrated by two ratters who were previously trained in the administration and scoring of the instrument. Ratters had access to the clinical chart of individuals recruited from the BPD Clinic of the INPRF.

Structured Clinical Interview for DSM-IV Personality Disorders (SCID-II) [26]: the presence of predominant narcissistic or antisocial personality traits was determined using the SCID-II, a semi-structured clinical interview with 119 items scored on a 3-point Likert scale (1 = absent to 3 = threshold), with additional exploratory questions the interviewer may use to score a particular item. For the present study, inter-rater reliability was determined, with kappa values greater than 0.75 (C.I. = 0.76–0.88) [28]. This interview was administrated by two clinicians who were blind to the PCL-R results.

Temperament and Character Inventory-Revised (TCI-R): a self-report questionnaire comprising 240-items scored on a 5-point Likert scale (1 = definitely false to 5 = definitely true) that assesses the four dimensions of temperament and the three of character [29]. The TCI-R has adequate validity and reliability (Cronbach alpha values > 0.80) in the Mexican population [30].

The complete evaluation lasted approximately two hours, and could be completed in one or two sessions according to the participant’s availability.

### 2.3. Statistical Analyses

Means, standard deviations and ranges were calculated for continuous variables while frequencies and percentages were calculated for categorical variables. Contingency table Chi-square tests were used to test differences among groups for categorical variables and univariate ANOVAs while Bonferroni tests were applied for continuous variables. A multiple, stepwise discriminant analysis was performed to evaluate the optimal combination of the temperament and character dimensions that are able to discriminate between individuals with psychopathy with predominant antisocial or predominant narcissistic personality traits and a control group. The probability value (*p*) of <0.05 was chosen as the level of statistical significance for all tests. All analyses were performed using IBM SPSS 21.0.

## 3. Results

### 3.1. Demographic Characteristics

Male individuals accounted for 51.3% of the subjects with psychopathy and control group (*n* = 41 in each group). The mean age of both groups was 32.9 years (S.D. = 10.6, range 18–57), while 57.6% (*n* = 46) of each group held at least a bachelor’s degree.

In accordance to the clinical interview performed at the BPD clinic prior to the recruitment of subjects, 93.8% (*n* = 75) of the individuals with psychopathy had a DSM-5 Axis-I disorder. Attention deficit disorder was the most frequent diagnosis (52.2%, *n* = 39) followed by substance abuse/dependence (29.3%, *n* = 22), major depression (13.3%, *n* = 10), and bulimia (5.3%, *n* = 4).

Half the individuals with psychopathy (*n* = 40) were classified in the group with predominant antisocial personality traits (PsyAP) while the other half reported predominant narcissistic personality traits (PsyNP). The PCL-R scores were significantly higher in individuals with psychopathy compared to the control group (*p* < 0.001). In addition, individuals from the PsyAP group reported higher scores in the PCL-R Factor 2—lifestyle and antisocial (Bonferroni <0.001) and the PCL-T Total score (Bonferroni <0.001) compared to the PsyNP individuals (Table 1).

### 3.2. Discriminant Analysis Using Temperament and Character Dimensions

The Kolmogorov–Smirnov test showed normal distribution of TCI-R scores in each group with *p*-values > 0.05 (control group range: 0.45–1.04; PsyAP group range: 0.52–0.97; and PsyNP group range: 0.6–0.86).

**Figure 1:**
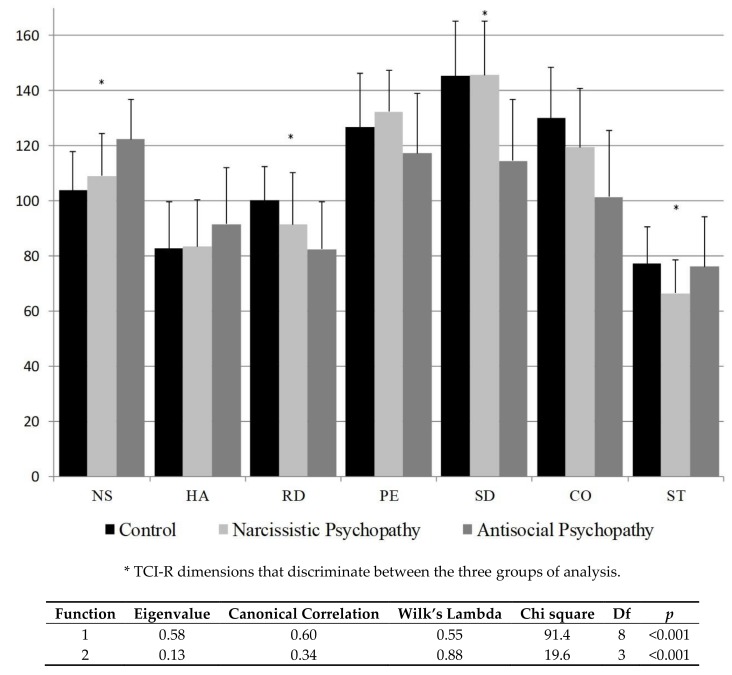
Temperament and character dimensions between control, antisocial psychopathy, and narcissistic psychopathy groups.

The mean scores and standard deviations of the temperament and character dimensions of the three groups are shown in Figure 1. Significant differences emerged between the three groups in all temperament and character dimensions. PsyAP individuals differed from control subjects and PsyNP subjects—both with similar scores with Bonferroni >0.05—with higher novelty seeking (NS, Bonferroni *p* < 0.001) and harm avoidance (HA, Bonferroni <0.05) and lower persistence (PE, Bonferroni <0.05) in the temperament dimensions and lower self-directedness (SD, Bonferroni *p* < 0.001) in the character dimensions. Reward dependence (RD) and cooperativeness (CO) differ between the three groups (Bonferroni *p* < 0.05), with PsyAP reporting lower scores and the control group having higher scores. Self-transcendence (ST) was similar between the control group and PsyAP but differed significantly from the PsyNP group (Bonferroni *p* < 0.05).

Two discriminant functions were extracted (Function 1: Wilkin’s Lambda = 0.55, *p* < 0.001 and Function 2: Wilkin’s Lambda = 0.88, *p* < 0.001), both with significant canonical correlations (0.62 and 0.34, respectively) to distinguish between the three groups analyzed. The first function clearly distinguishes the PsyAP group (centroid value −1.2) from the PsyNP and control group (centroid values 0.2 and 0.5, respectively) with self-directedness (SD), novelty seeking (NS), and reward dependence (canonical discriminant standardized functions = 0.60, −0.43, and 0.32, respectively) being the most important discriminant variables. Although the second function was significant for distinguishing the PsyNP group (centroid value −0.6) from the other groups (PsyAP centroid value 0.1 and control group centroid value 0.2), the Wilkin’s Lambda value exhibits an overlap of variables (PsyNP and control groups). Self-transcendence (ST), reward dependence (RD), and self-directedness (SD; canonical discriminant standardized functions = 0.86, 0.43, and −0.34, respectively) were the most important personality dimensions for discriminating between the groups.

## 4. Discussion

The aim of the present study was to compare temperament and character dimensions between individuals with psychopathy with comorbid predominant antisocial or narcissistic personality traits and control subjects and to determine which dimensions distinguish these groups.

Our hypotheses were partially confirmed as discriminant analysis results showed that individuals with psychopathy might be distinguished by some of their temperament and character traits and that more similarities were observed between PsyNP and control subjects. However, we hypothesized that cooperativeness would be one of the dimensions able to discriminate between groups, but this was not supported by our results. Other dimensions, specifically reward dependence and self-transcendence, were important dimensions that distinguish between groups.

As previously stated, antisocial personality traits have been extensively associated with psychopathy. However, there have also been cross-references citing narcissism as a construct related to psychopathy [12].

When comparing demographic features between our groups, we observed that control subjects and PsyNP were similar in terms of occupation and educational attainment, which are usually seen as indicators of social achievement and were less frequently observed in the PsyAP group. Although both groups with psychopathy report similar interpersonal/affective scores in the PCL-R Factor 1, PsyNP displayed fewer aspects of antisociality (lower scores in the lifestyle/antisocial Factor 2), which may make them appear more functional or even to have abilities and talents that can be advantageous for their adaptation in their social environment [31]. Some authors consider that there is a close correspondence between psychopathy and narcissism traits [32] and that this association may depend on the measurement used for both constructs [33,34].

In personality theory, making a clear distinction between the source and its expression is no easy task. The difficulty is exacerbated by the likelihood that both are linked to some of the same underlying psychobiological and developmental processes [2] and should be studied further in terms of a dimensional model of personality like the one used in the present study. Even though differences in the temperament and character dimensions were observed between the three groups of analysis in our study, only two temperament dimensions (novelty seeking and reward dependence) and two character dimensions (self-directedness and self-transcendence) were relevant for discriminating between the groups.

Novelty seeking is the most frequently reported dimension associated with the classic description of psychopathy [35,36,37], particularly in terms of antisocial behaviors such as impulsivity, reckless behavior, need for novel stimuli, and poor behavioral control. In our research, NS was the most important personality dimension of PsyAP since the remaining groups (PsyNP and control) achieved similar scores.

The other temperament dimension that marked a difference was reward dependence, which was associated with a tendency to respond to behavior and rewards [19]. Since it includes sentimentality, openness to communication, attachment, and dependence, it is hardly surprising that scores in this dimension were lower in the PsyAP group. On the other hand, for the PsyNP group, one described feature of narcissism is its apparent dependence on reward [38]. Since these individuals may be less impulsive and reckless, some of their psychopathic expressions (such as charm, charisma, and manipulation) may be aimed to obtain gratification, social approval, and support.

In general, difficulties in any character dimensions are associated with the presence of personality disorder traits [38]. Lower levels of self-directedness are associated with blaming others, immature, childish behavior and reactivity in social behavior; while higher levels are associated with successful leadership, self-esteem, the sensation of purpose or meaning in their lives, and the capacity to postpone desires to achieve goals. These descriptions virtually describe the traits of antisociality and narcissism without psychopathy [4,5]. In fact, it would appear that individuals with PsyNP are better adapted to society than those with PsyAP, even though interpersonal difficulties associated with psychopathy are present. Nevertheless, the role of self-directedness dimension in the expression of psychopathic traits should not be overlooked as psychopathy represents the conjunction of personality traits and behaviors [2]. Therefore, as a personality dimension conditioned by life experiences and environment, this dimension, in conjunction with the presence of specific personality disorders, may shape the way psychopathic traits are expressed.

On the other hand, in the words of Cloninger [19] (p. 270), the self-transcendence dimension: “involves the spontaneous feeling of participation in one’s surrounding as a unitive whole”. This dimension is more complex than the other character dimensions as it involves abstraction, self-awareness, spirituality, and rational materialism associated with wise judgment. “Individuals who are highly transcendent often report frequent periods of joyful unity and creative inspiration that they do not attribute to self-directed analysis” [39] (p. 270). In our analysis, PsyAP subjects show the same levels in this dimension as control subjects, suggesting that those with PsyAP have more abstraction, transpersonal identification, and spirituality than those with PsyNP.

Given that PsyNP report higher self-directedness, it would appear they have more control over their social behavior for achieving their goals [1] but are more distant in their relationships with others. This is paradoxical, because PsyAP subjects may commit crimes and exhibit aggressive behavior, both of which are unacceptable for any society. We hypothesize that higher self-transcendence observed in PsyAP individuals may be the reflection of the continuous need to belong [40] related to previous experiences of social exclusion or rejection [41]. This need may be constantly threatened by antisocial behaviors and psychopathy [42] and the response to this threat is expressed through the maintenance of these behaviors, which in turn reduces the likelihood of securing the desired social acceptance [40,42]. Their high ST and ongoing frustration associated with rejection may elicit a range of negative consequences. PsyAP may fulfill their need to belong by becoming members of gangs, organized crime and other criminal associations, where their behavior may be perceived as congruent and in line with the codes of behavior of these groups (for example, the rules of a criminal organization).

Our results provide further support for Patrick’s triarchic psychopathy construct [16,43,44] involving disinhibition, meanness, and boldness. The basic part of disinhibition may be associated with the temperament dimension of novelty seeking, which was more evident for respondents in the PsyAP group. The second and third elements of the psychopathy construct, meanness, and boldness were both evaluated through Factor 1 of the PCL-R with similar scores in both groups. Meanness includes “callousness, cold-heartedness and antagonism, … agentic disaffiliation, …, arrogance and verbal derisiveness, …” [16] (p. 926). While boldness refers to “a capacity to remain calm and focused in situations involving pressure or threat, an ability to recover quickly from stressful events, high self-confidence and social efficacy, and tolerance for unfamiliarity and danger” [16] (p. 926). From this perspective, as Patrick points out, the interaction of disinhibition and meanness suggests a difficult temperament (in our research, high novelty seeking and low reward dependence in those with PsyAP) while the interaction of meanness and boldness expresses a lack of fear (in our results, the same rates in Factor 1 of the PCL-R in both groups).

Lastly, with the Dark Triad Theory of psychopathy [21], García and Rosenberg [22], found that a high Machiavellianism is related to lower self-directedness and cooperativeness, a profile that is similar to what we observed in the PsyAP group. According to this theory, subjects with high narcissism also reported higher self-directedness as we found in our PsyNP group.

Our study had some limitations worth considering. The small sample size of the psychopathy groups limits the generalization of our results. Since personality is the result of biology and environment, other key variables should be included to determine whether psychopathic disorders can be defined in accordance to the predominance of personality traits as antisocial or narcissistic. These variables include the type of parenting received, attachment, psychiatric history in childhood, evaluation of the social environment and substance use. Another possible limitation was the presence of some Axis-I disorders, in particular attention deficit disorder and substance abuse/dependence. Both diagnoses might have a direct influence on the overt expression of antisocial behavior as attentional deficits and difficulty in decision-taking are related to these diagnoses [45,46].

## 5. Conclusions

Our results gave further support of the existence of several psychopathic disorders as reported in the Dark Triad Theory and the Triarchic Psychopathy Construct that might be identified in accordance to personality traits. Moreover, the identification of different psychopathic disorders should be theoretically viable, applicable to events observed in our everyday lives and useful for identifying the care strategies each subject requires for their adequate social integration and individual well-being. For example, interventions aimed to reduce or eradicate antisocial behavior for one and improving interpersonal relationships for the other. The present research also supported the existence of a successful psychopathy, more related to personality traits commonly seen in the general population, which favors the evidence of the need to reduce stigma related to psychopathy, where individuals are frequently stigmatized as incurable and dangerous.

## Figures and Tables

**Table 1:** Demographic and clinical characteristics between individuals with psychopathy with comorbid antisocial or narcissistic personality disorder.

	Control Group *n* = 80	Antisocial Psychopathy *n* = 40	Narcissistic Psychopathy *n* = 40	Statistics
		*n* %	*n* %	
*Gender*				
Male	41 51.2	23 57.5	18 45.0	χ^2^ = 1.2, *p* = 0.53
Female	39 48.8	17 42.5	22 55.0	
*Occupation*				
None	1 1.3	16 40.0	3 7.5	
Housewife	6 7.5	1 2.5	2 5.0	χ^2^ = 40.3,
Student	16 20.0	9 22.5	9 22.5	*p* < 0.001
Employed	57 71.3	14 35.0	26 65.0	
*Educational Achievement*	34 42.5	31 77.5	3 7.5	χ^2^ = 40.1,
High school or less	46 57.5	9 22.5	37 92.5	*p* < 0.001
Bachelor’s degree or higher				
	**Mean; SD; Range**	**Mean; SD; Range**	**Mean; SD; Range**	
*Age*	32.9; 10.6; 18–57	30.3; 10.2; 18–57	35.5; 10.5; 20–57	F = −2.2, *p* = 0.08
*PCL-R*				
Factor 1	2.3; 2.2; 0–9	14.6; 1.7; 10–16	15.0; 1.0; 13–16	F = 908.7, *p* < 0.001
Factor 2	2.0; 1.5; 0–5	14.9; 2.5; 10–20	9.9; 2.0; 7–15	F = 637.7, *p* < 0.001
Total	4.2; 3.3; 0–13	31.4; 3.5; 25–39	35.5; 10.5; 20–57	F = 1239.7, *p* < 0.001

A comparison of demographic characteristics (Table 1) showed that those with PsyAP were more likely to be unemployed and have lower educational attainment than those with PsyNP and control subjects.
